# Free Radical-Scavenging, Anti-Inflammatory/Anti-Fibrotic and Hepatoprotective Actions of Taurine and Silymarin against CCl_4_ Induced Rat Liver Damage

**DOI:** 10.1371/journal.pone.0144509

**Published:** 2015-12-11

**Authors:** Ashraf M. Abdel-Moneim, Mohammed A. Al-Kahtani, Mohamed A. El-Kersh, Mohammed A. Al-Omair

**Affiliations:** 1 Department of Biological Sciences, Faculty of Science, King Faisal University, Al-Hassa, Saudi Arabia; 2 Department of Zoology, Faculty of Science, Alexandria University, Alexandria, Egypt; 3 Department of Chemistry, Faculty of Science, King Faisal University, Al-Hassa, Saudi Arabia; 4 Department of Biochemistry, Faculty of Science, Alexandria University, Alexandria, Egypt; University of Navarra School of Medicine and Center for Applied Medical Research (CIMA), SPAIN

## Abstract

The present study aims to investigate the hepatoprotective effect of taurine (TAU) alone or in combination with silymarin (SIL) on CCl_4_-induced liver damage. Twenty five male rats were randomized into 5 groups: normal control (vehicle treated), toxin control (CCl_4_ treated), CCl_4_+TAU, CCl_4_+SIL and CCl_4_+TAU+SIL. CCl_4_ provoked significant increases in the levels of hepatic TBARS, NO and NOS compared to control group, but the levels of endogenous antioxidants such as SOD, GPx, GR, GST and GSH were significantly decreased. Serum pro-inflammatory and fibrogenic cytokines including TNF-α, TGF-β1, IL-6, leptin and resistin were increased while the anti-inflammatory (adiponectin) cytokine was decreased in all treated rats. Our results also showed that CCl_4_ induced an increase in liver injury parameters like serum ALT, AST, ALP, GGT and bilirubin. In addition, a significant increase in liver tissue hydroxyproline (a major component of collagen) was detected in rats exposed to CCl_4_. Moreover, the concentrations of serum TG, TC, HDL-C, LDL-C, VLDL-C and FFA were significantly increased by CCl_4_. Both TAU and SIL (i.e., antioxidants) post-treatments were effectively able to relieve most of the above mentioned imbalances. However, the combination therapy was more effective than single applications in reducing TBARS levels, NO production, hydroxyproline content in fibrotic liver and the activity of serum GGT. Combined treatment (but not TAU- or SIL-alone) was also able to effectively prevent CCl_4_-induced decrease in adiponectin serum levels. Of note, the combined post-treatment with TAU+SIL (but not monotherapy) normalized serum FFA in CCl_4_-treated rats. The biochemical results were confirmed by histological and ultrastructural changes as compared to CCl_4_-poisoned rats. Therefore, on the basis of our work, TAU may be used in combination with SIL as an additional adjunct therapy to cure liver diseases such as fibrosis, cirrhosis and viral hepatitis.

## Introduction

Carbon tetrachloride (CCl_4_) is a potent hepatotoxin widely used for induction of chemical liver damage involving the aggravation of inflammatory processes and recruitment of inflammatory cells [1,2]. The toxicity of CCl_4_ is attributed to the reactive oxygen species (ROS) and free radicals produced during its metabolism [3]. Several hepatoprotective agents, including natural substances from medicinal plants have been reported to counteract ROS-mediated tissue damage by their antioxidant and free radical scavenging abilities [4–9].

Taurine (2-amino ethane sulphonic acid; TAU) a nonproteinogenic sulfur containing-amino acid, has been reported to have a cytoprotective role [10]. TAU is known to improve cellular antioxidant defense system, stabilize biomembranes and reduce *in vivo* lipid peroxidation (LPO), thus preventing apoptosis and necrotic cell death [11–13]. TAU supplementation have been also shown to attenuate steatosis and hepatotoxicity in several animal models [14–18]. Silymarin (SIL), a polyphenolic flavonoid confined from milk thorn is another antioxidant that has been also proven to protect against liver injuries induced by various hepatotoxins, including CCl_4_ [19–22]. SIL increases the activity of nucleolar polymerase A, with subsequent increment in ribosomal protein synthesis, in this way invigorating the regenerative capacity of the liver and the formation of new hepatocytes [23,24]. Furthermore, it maintains the integrity of the hepatocyte cellular membrane and prevents the entrance of liver toxins or xenobiotics [25]. Due to its phenolic nature, SIL also prevents lipoperoxidation of membranes and scavenges ROS, thus increasing GSH availability [24,25]. This study is the first to demonstrate the combined effects of TAU and SIL on CCl_4_-mediated hepatotoxic insult in male rats and to compare such effects to their respective individual effects. For this purpose we evaluated indices of oxidative/nitrosative stress, several inflammatory molecules, markers of liver function tests, lipid profile and histomorphological changes.

## Materials and Methods

### Drugs and chemicals

CCl_4_, TAU and SIL were purchased from Sigma Chemical Company, USA. Other chemical reagents were of high-quality analytical grade. CCl_4_ was diluted with olive oil while TAU and SIL solutions were prepared in 0.1 M phosphate buffer saline with pH 7.4.

### Animals and treatments

**Fig 1:**
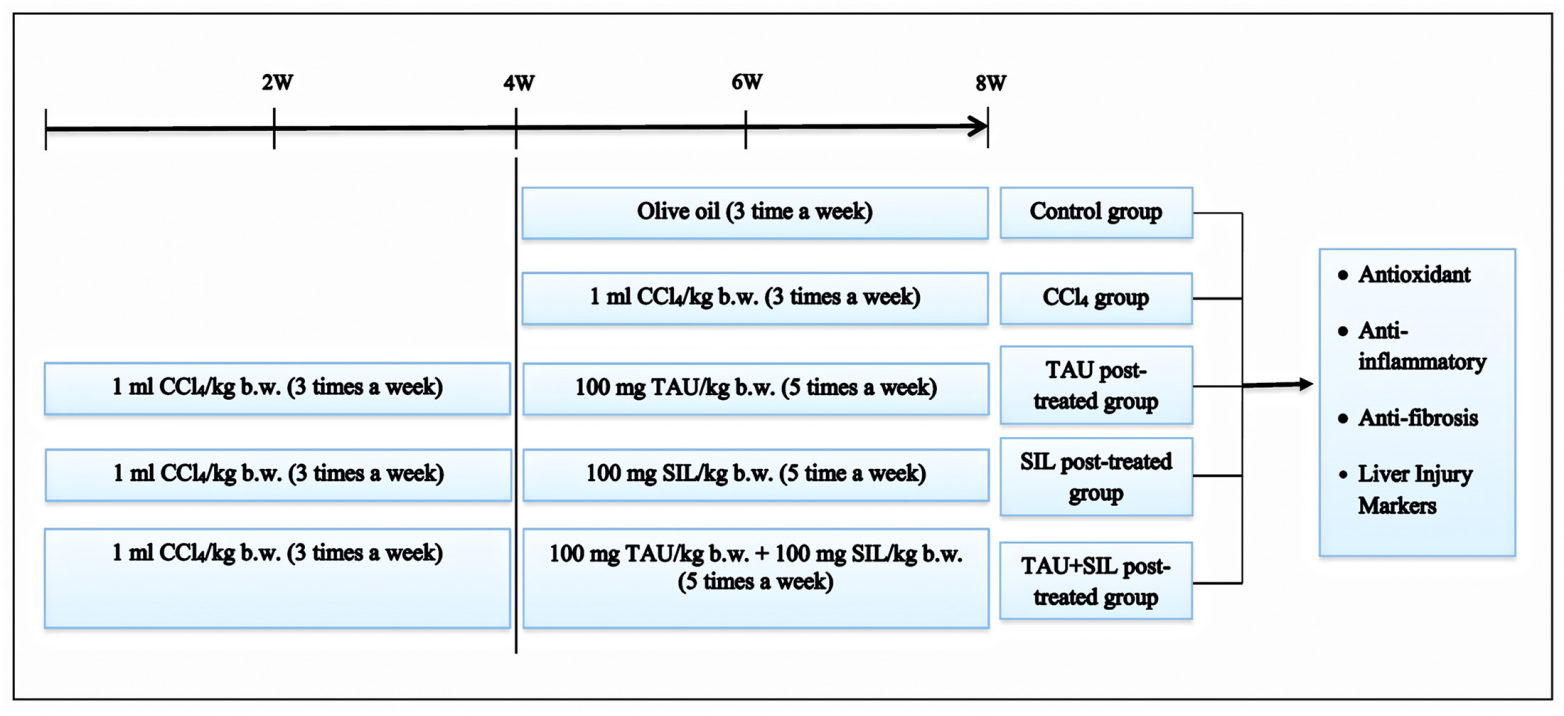
Schematic diagram of *in vivo* experimental protocol.

Institutional Animal Care and Use Committee (IACUC) at the King Faisal University approved the experimental protocol of this study. Healthy adult male Wister albino rats (155–190 g) were obtained from animal house facility at King Saud University, Saudi Arabia. All rats were housed in polyethylene cages under controlled laboratory conditions and provided with standard rat chow and water *ad libitum*. They were allowed 1-week acclimatization period before the initiation of the experiment. Fig 1 summarizes the animals grouping and their treatment process. In brief, rats were randomly assigned into 5 groups (n = 5 per group). Group 1 (normal control) was given 0.5 ml olive oil/kg intraperitoneally (i.p.) 3 times a week for 4 weeks. Group 2 (toxin control) received 1 ml CCl_4_ (50% in olive oil)/kg b.w. (i.p.) 3 times a week for 4 weeks. Group 3 received CCl_4_ as group 2 followed by 100 mg TAU/kg b.w. (i.p.) 5 times a week for 4 weeks more. Group 4 was given CCl_4_ as group 2 followed by 100 mg SIL/kg b.w. (i.p.) 5 times a week for 4 weeks more. Group 5 received CCl_4_ as group 2 followed by 100 mg TAU/kg b.w. and 100 mg SIL/kg b.w. (i.p.) 5 times a week for 4 weeks more. The doses of TAU and SIL were selected based on previous investigations of Flora et al. [26] and Shaker et al. [27], respectively.

After 24 h of the last dose, the rats were anesthetized under light ether, and all efforts were made to minimize suffering and stress. After laparotomy, samples of trunk blood were collected from the abdominal aorta and centrifuged at 5000 rpm for 10 min; the separated sera were stored frozen until analysis. In addition, livers were removed quickly and homogenized with Glass Col homogenizer, and a 20% w/v homogenate was prepared in ice cold PBS (50 mM, pH 7.4). The homogenate was centrifuged at 5000 rpm for 20 min, and the supernatant was divided over several vials to avoid sample thawing and freezing and was kept at -80°C till processed. Samples of intact liver tissues were used for light and electron microscopic studies.

### Assessment of antioxidants, lipid peroxidation and nitrogen radicals

Biochemical markers of oxidative and nitrosative stress were detected in the supernatant of liver homogenate. Superoxide dismutase (SOD, EC 1.15.1.1) was estimated as previously reported by Ukeda et al. [28]. The activity of glutathione peroxidase (GPx, EC 1.11.1.9) was measured by the method of Jacobson et al. [29]. Glutathione reductase (GR, EC 1.6.4.2) was assayed following the method described by Carlberg and Mannervik [30]. Glutathione -S-transferase (GST, EC 2.5.1.18) was measured based on the method of Habig et al. [31]. The concentration of reduced glutathione (GSH) in liver was determined using the method of Lindenmaier et al. [32]. Lipid peroxidation (LPO) was determined by measuring thiobarbituric acid reactive substances (TBARS) according to the method of Ohkawa et al. [33]. Nitric oxide (NO) level was estimated based on the addition of Griess reagent to nitrite [34]. Nitric oxide synthase (NOS, EC 1.14.13.39) assay was performed using Griess reaction for the colorimetric measurement of total nitrite, which is the final product of NO formed with the catalyzing role of NOS in aqueous solution [35]. Total protein content was estimated by the method of Lowry et al. [36] using bovine serum albumin as standard.

### Measurement of serum cytokines

A panel of specific cytokines, namely adiponectin, tumor necrosis factor-alpha (TNF-α), transforming growth factor-beta1 (TGF-β1), interleukin-6 (IL-6), leptin and resistin was tested using appropriate ELISA kits. Adiponectin and TNF-α ELIZA kits were obtained from RayBiotech, Inc. (Norcross, GA, USA) and TGF-β1, IL-6 and leptin kits from Aviscera Bioscience, Inc. (Santa Clara, CA, USA). ELIZA assay kit (Biorbyt, Cambridgeshire, UK) was used to measure resistin.

### Determination of biochemical markers of hepatic injury

Serum levels of alanine aminotransferase (ALT; EC 2.6.1.2), aspartate aminotransferase (AST; EC 2.6.1.1), alkaline phosphatase (ALP; EC 3.1.3.1) and γ-glutamyltransferase [(γ-glutamyl)-peptide: amino acid γ-glutamyltransferase (GGT; EC 2.3.2.2)] and total bilirubin were determined according to the instructions supplied with the assay kits (Human Gesellschaft für Biochemica und Diagnostica mbH, Germany). Hepatic hydroxyproline was measured using commercial diagnostic kit (BioVision Research Products, Linda Vista Avenue, Mountain View, CA, USA).

### Estimation of serum lipid profile parameters

Triglycerides (TG), total cholesterol (TC) and high density lipoprotein-cholesterol (HDL-C) were assayed by enzymatic colorimetric test using human kits (Germany) according to the manufacturer protocol. Low-density lipoprotein (LDL-C) and very low-density lipoprotein-cholesterol (VLDL-C) were calculated using Friedewald’s equations [37]. Serum free fatty acids (FFA) level was measured by the method of Jelinek et al. [38].

### Histopathological analysis of liver lesions

Liver specimens were fixed in 10% formalin solution and processed routinely for paraffin embedding. Sections (4 μm-thick) were deparaffinized and then stained with haematoxylin and eosin solutions (H&E), and examined under light microscope (Nikon 80i, Japan). To reveal collagen fibers, fixed sections were stained with Masson trichrome methods. All sections were evaluated for the degree of liver injury. Each liver slide was examined and assigned for severity of changes using scores on a scale of 0 –Absent; +–few; ++–mild; +++–moderate; ++++–severe; +++++–extremely severe.

### Electron microscopic investigations

Small slices of liver were fixed in 3% glutaraldehyde in sodium phosphate buffer (200 mM, pH7.2) for 3 h at 4°C. Materials were washed in the same buffer and postfixed in cold 1% osmium tetroxide (Agar Sci. Ltd., England) for 1 h. After flushing in phosphate buffer, the tissue samples were dehydrated in graded ethanol solutions and embedded in Araldite (Agar Sci. Ltd., England). Thin sections (80–100 nm) were cut using Leica EM UC6 (Leica Co., Austria) ultramicrotome. Sections were mounted on grids and double stained with 2% uranyl acetate and lead citrate. Sections were viewed and photographed on Jeol JEM 1011 transmission electron microscope (Jeol Ltd., Japan) at 80 kV.

### Statistical analysis

The data were analyzed using SPSS 17.0 for windows. All variables were compared using one-way analysis of variance (ANOVA) followed by LSD post hoc test. P≤0.05 was considered statistically significant.

## Results

### Biochemistry

Biochemical variables indicative of free radical injury are shown in Table 1. A significant increase was observed in the hepatic TBARS, NO and NOS after CCl_4_ treatment compared to normal control. In contrast, a significant decline in SOD, GPx, GR, GST and GSH was found in the liver of CCl_4_-treated animals. Post-treatment with TAU-alone alleviated TBARS levels without reaching normal values, while SIL-alone and TAU+SIL restored TBARS to normalcy, with maximum reduction recorded after TAU+SIL treatment. Similarly, the combination of TAU+SIL elicited a more intense reduction in CCl_4_-induced elevation of liver NO level and NOS activity compared to individual treatments, yet, the level of NO was still somewhat different from the control. In the meantime, the inhibited activities of SOD, GPx and GR after treatment with CCl_4_ were significantly increased in rats post-treated with TAU, SIL and TAU+SIL compared to CCl_4_-alone group. SIL monotherapy (but not TAU-alone) and the combined treatment of TAU+SIL resulted in a significant increase in hepatic GST and GSH comparable to the hepatotoxic group.

**Table 1:** Liver oxidant-antioxidant status, NO levels and NOS activity.

Groups	SOD U/mg protein	GPx nmol/mg protein	GR mU/mg protein	GST mU/mg protein	GSH μmol/mg protein	TBARS nmol MDA/g tissue	NO nmol/mg protein	NOS nmol/min/mg protein
Control	4.81±0.40^a^	90.36±4.82^a^	1.64±0.11^a,b^	7.54±0.75^a^	15.13±0.86^a^	73.80±5.69^a,b^	1.61±0.09^a^	3.86±0.14^a^
CCl_4_	2.25±0.47^b^	54.40±1.39^b^	0.68±0.11^c^	3.60±0.48^b^	9.11±0.71^b^	149.55±3.67^c^	3.95±0.29^b^	7.82±0.88^b^
CCl_4_ + TAU	4.36±0.23^a^	90.68±12.81^a^	1.24±0.12^a^	5.01±0.54^b,c^	12.73±0.78^a,b^	99.11±13.03^d^	3.15±0.44^c^	6.02±0.76^c^
CCl_4_ + SIL	5.39±0.63^a^	102.40±5.23^a^	1.48±0.26^a,b^	6.12±0.66^a,c^	13.85±1.34^a^	84.26±13.93^a,d^	3.12±0.19^c^	6.21±0.51^c^
CCl_4_ + TAU + SIL	6.40±0.56^a^	105.04±10.91^a^	1.73±0.16^b^	6.40±0.49^a,c^	14.92±1.40^a^	63.87±6.10^b^	2.57±0.10^d^	4.67±0.30^a^

SOD, superoxide dismutase; GPx, glutathione peroxidase; GR, glutathione reductase; GST, glutathione-s-transferase; GSH, reduced glutathione; TBARS, thiobarbituric acid reactive substances; MDA, malondialdehyde; NO, nitric oxide; NOS, nitric oxide synthase.

Results are means±SEM of five replicate determinations.

Values within a column not sharing common superscript letters are significantly different at P≤0.05.

As shown in Table 2, high levels of pro-inflammatory cytokines (i.e., TNF-α, TGF-β1, IL-6, leptin and resistin) were detected in serum of all rats after CCl_4_ treatment. In addition, CCl_4_ caused a significant decline in serum concentrations of the anti-inflammatory adipocyte-derived adiponectin. The elevated levels of TGF-β1, IL-6 and leptin induced by CCl_4_ were reduced after treatment with TAU, SIL and TAU+SIL but TGF-β1 and IL-6 were still significantly higher than those of healthy controls. SIL-alone did not significantly reduce TNF-α and resistin levels which responded favorably to both TAU-alone and TAU+SIL post-treatments in comparison to CCl_4_ control group. Interestingly, only the combination of TAU+SIL (but not the individual drugs) efficiently restored the inhibited adiponectin levels to near normal.

**Table 2:** Serum cytokine levels.

Groups	Adiponectin pg/ml	TNF-α pg/ml	TGF-β1 pg/ml	IL-6 pg/ml	Leptin ng/ml	Resistin pg/ml
Control	8.34±0.40^a^	51.22±7.18^a^	210.47±17.11^a^	37.67±2.84^a^	1.81±0.22^a,b^	118.00±10.90^a^
CCl_4_	5.35±0.41^b^	106.70±8.29^b^	434.68±16.03^b^	113.50±9.56^b^	2.82±0.24^c^	195.50±16.65^b^
CCl_4_ + TAU	5.86±0.41^b^	83.73±6.21^c^	339.53±36.53^c^	66.00±7.10^c^	2.03±0.31^b^	134.88±10.53^a^
CCl_4_ + SIL	5.81±0.50^b^	93.22±7.08^b,c^	283.88±16.00^c,d^	83.17±7.66^d^	1.70±0.16^a,b^	163.00±13.01^a,b^
CCl_4_ + TAU + SIL	7.54±0.89^a^	79.64±8.13^c^	269.35±26.25^d^	57.50±8.69^c^	1.26±0.16^a^	120.50±8.40^a^

TNF-α, tumor necrosis factor-alpha; TGF-β1, transforming growth factor-beta1; IL-6, interleukin-6.

Results are means±SEM of five replicate determinations.

Values within a column not sharing common superscript letters are significantly different at P≤0.05.

The hepatoprotective, curative effects of TAU and SIL singly or in combination, on biochemical markers of hepatic injury in CCl_4_ intoxicated rats are shown in Table 3. Rats treated with CCl_4_ had significantly elevated levels of serum ALT, AST, ALP, GGT, and total bilirubin compared to normal control group. Likewise, the hepatic hydroxyproline in CCl_4_ treated rats was significantly higher than the normal levels. However, treatment with TAU, SIL and their combination after CCl_4_ intoxication significantly reduced the elevated levels of ALT, AST, ALP, GGT, total bilirubin, and hydroxyproline compared to the hepatotoxin treatment group. Notably, post-treatment with SIL-alone or in combination with TAU reduced ALT, AST, and total bilirubin towards normalization whereas ALP activity was normalized by TAU-alone and TAU+SIL treatments. It may also be worth noting that the combination of TAU+SIL improved the reversal of serum GGT activity and liver hydroxyproline levels over that by either of them alone.

**Table 3:** Biochemical markers of hepatic injury.

Groups	ALT U/l	AST U/l	ALP U/l	GGT U/l	Total Bilirubin mg/dl	Hydroxyproline mg/g tissue
Control	11.97± 1.76^a^	41.90± 2.60^a^	136.29±13.74^a^	5.83±0.73^a^	2.50±0.54^a^	3.50±0.49^a^
CCl_4_	51.36± 6.28^b^	141.86±12.04^b^	249.75±11.18^b^	37.48±2.55^b^	4.97±0.24^b^	17.33±1.66^b^
CCl_4_ + TAU	37.26±11.09^c^	69.64±11.66^c^	146.33±13.97^a^	30.20±2.14^c^	3.72±0.19^c^	13.02±0.78^c^
CCl_4_ + SIL	18.88± 1.04^a^	59.74± 1.83^a,c^	197.40±13.65^c^	24.16±1.42^d^	3.44±0.23^a,c^	12.34±0.35^c^
CCl_4_ + TAU + SIL	19.11± 2.78^a^	59.51± 7.82^a,c^	164.44±14.75^a,c^	20.61±1.67^e^	3.03±0.29^a,c^	9.52±0.75^e^

ALT, alanine aminotransferase; AST, aspartate aminotransferase; ALP, alkaline phosphatase; GGT: γ-glutamyltransferase.

Results are means±SEM of five replicate determinations.

Values within a column not sharing common superscript letters are significantly different at P≤0.05.

The alterations in the levels of serum lipids in normal and experimental rats are presented in Table 4. TG, TC, HDL-C, LDL-C, VLDL-C and FFA were highly increased in CCl_4_ group compared to normal control. Levels of TG, TC and VLDL-C in TAU post-treated group were significantly decreased when compared to CCl_4_ solely treated group. Also, TG, TC, HDL-C, LDL-C and VLDL-C levels were down-regulated in SIL and TAU+SIL post-treated groups, with normal levels of TG, LDL-C and VLDL-C. After administration of CCl_4_, the levels of FFA in rats given separate treatments of TAU or SIL were the same as those treated with CCl_4_ alone, while FFA levels were restored to almost normal values when rats were given TAU+SIL.

**Table 4:** Serum lipid profile.

Groups	TG mg/dl	TC mg/dl	HDL-C mg/dl	LDL-C mg/dl	VLDL-C mg/dl	FFA mM
Control	77.80±6.94^a^	89.29±5.34^a^	53.60±3.61^a^	28.87±7.27^a^	15.56±1.39^a^	1.34±0.20^a^
CCl_4_	139.79±11.46^b^	173.95±18.09^b^	85.57±3.96^b^	60.42±13.76^b^	27.96±2.29^b^	2.94±0.53^b^
CCl_4_ + TAU	110.63±10.41^c^	140.16±10.89^c^	74.65±5.09^b,c^	50.67±8.75^b,c^	22.13±2.08^c^	2.12±0.49^b,c^
CCl_4_ + SIL	60.68±3.47^a^	119.81±2.85^d^	70.79±6.05^c^	36.884.68^a,c^	12.14±0.70^a^	2.23±0.21^b,c^
CCl_4_ + TAU + SIL	78.14±10.50^a^	116.71±3.03^d^	67.87±4.57^c^	33.21±7.07^a^	15.63±2.10^a^	1.92±0.22^a,c^

TG, triglycerides; TC, total cholesterol; HDL-C, high density lipoprotein-cholesterol; LDL-C, low density lipoprotein-cholesterol; VLDL-C, very low density lipoprotein-cholesterol; FFA, free fatty acids.

Results are means±SEM of five replicate determinations.

Values within a column not sharing common superscript letters are significantly different at P≤0.05.

### Histological observations

**Fig 2:**
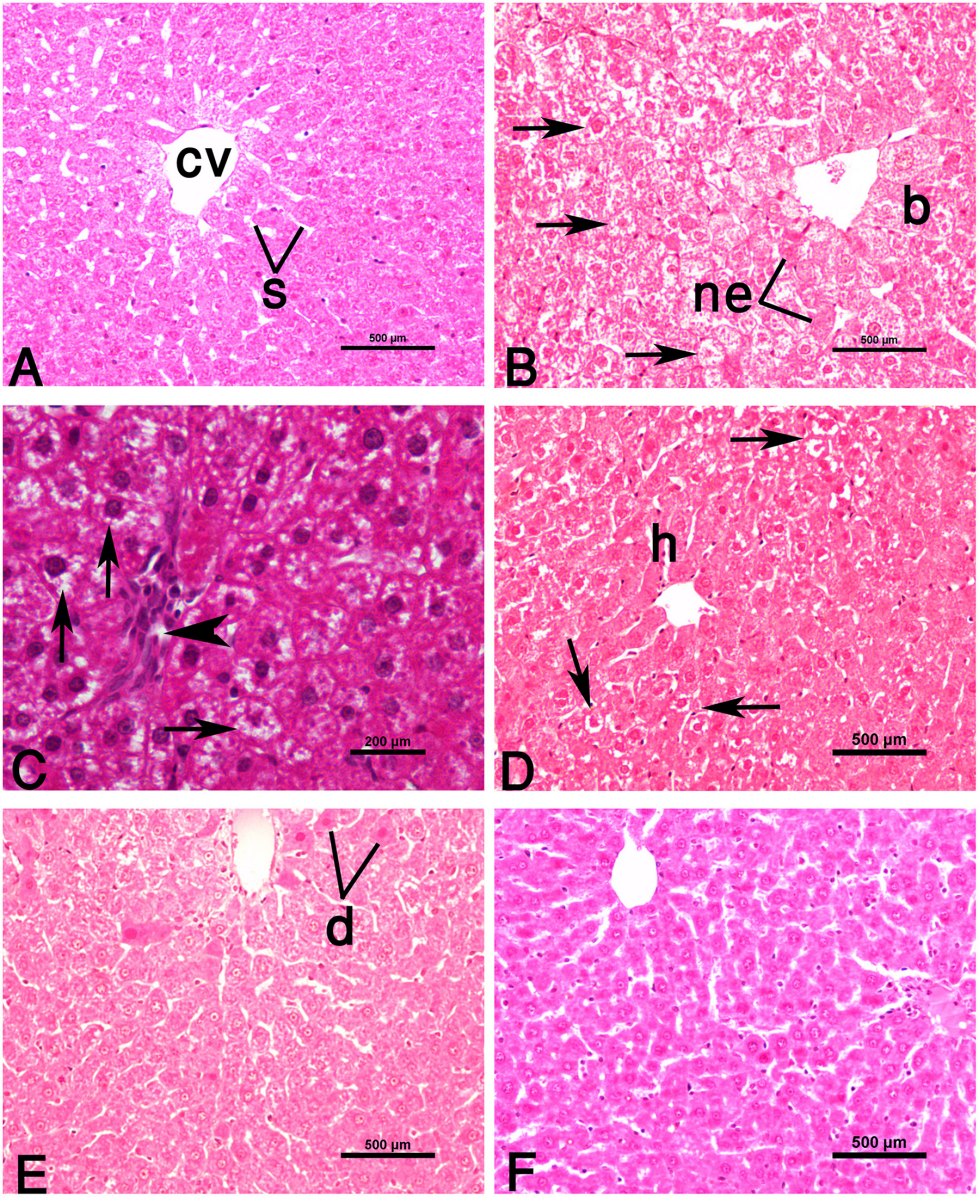
Photomicrographs of rat liver (H&E stain). **(A)** Control liver showing normal hepatocytes arranged in cords, obvious sinusoids (s), and central vein (cv). **(B, C)** Hepatic injury induced by CCl_4_. Note hepatic cells with ballooning degeneration (b), focal necrotic cell death (ne), and diffuse fatty changes (arrows) (in B). Microvesicular steatosis (i.e., accumulation of small fat droplets) in hepatocyte cytosol (arrows), and inflammatory infiltrates (arrowhead) are evident in liver tissue (in C). **(D)** CCl_4_+TAU group showing an improvement of cellular structure and uniform sinusoidal arrays (compared to B). Pathological fatty deposition (arrows) and also normal centrilobular hepatocytes (h) with well-defined cell borders, dense cytoplasm and central nuclei are visible. **(E)** CCl_4_+SIL group with less severe liver injury. Focal hepatocellular degeneration (d) is observed. **(F)** CCl_4_+TAU+SIL group indicating no pathologic lesions.

The histopathological changes are graded and summarized in Table 5. The liver of control rats exhibited normal lobular architecture with hepatocytes arranged in hepatic cords radiating from a central vein and separated by obvious blood sinusoids (Fig 2A). The individual hepatic cell was polyhedral in shape and had well-preserved acidophilic cytoplasm and prominent nucleus. Treatment of CCl_4_ alone caused notable lesions including deformed cord arrangement, ballooning degeneration of hepatocytes, condensed nuclei, and widespread hepatocellular necrosis (Fig 2B). Furthermore, fatty degeneration (microvesicular steatosis) and broad infiltration of inflammatory leukocyte cells were also frequently observed (Fig 2C). However, administration of TAU to CCl_4_-treated rats lessened the destruction in lobule structure (Fig 2D). The CCl_4_+SIL group demonstrated a large amount of alleviation in necrotic areas and steatosis compared with the toxin group (Fig 2E). Furthermore, the histological recovery appeared more superior when applying a combined TAU+SIL therapy (Fig 2F).

**Table 5:** Grading of the histopathological changes in the liver sections.

Groups	Hepatocyte ballooning	Hepatocyte necrosis	Fatty changes (Lipidosis)	Inflammatory cells infiltration	Portal fibrosis
Control	0	+	0	+	0
CCl_4_	+++	+++++	+++++	++++	+++
CCl_4_ + TAU	+	+++	+++	++	++
CCl_4_ + SIL	+	++	++	++	++
CCl_4_ + TAU + SIL	0	+	+	+	+

Scoring was done as follows: 0 –Absent; +–few; ++–mild; +++–moderate; ++++–severe; +++++–extremely severe.

**Fig 3:**
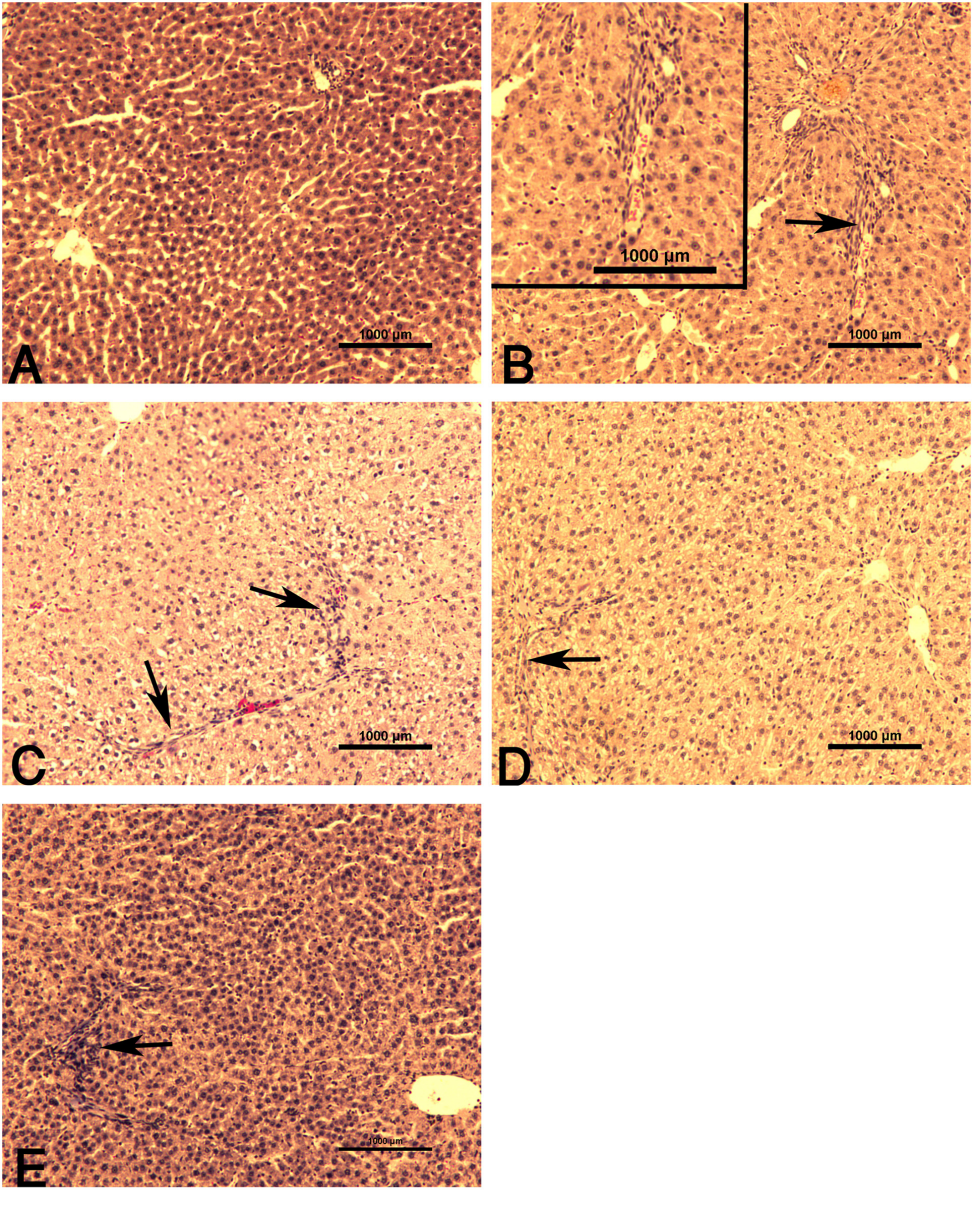
Photomicrographs of rat liver (Masson trichrome stain). **(A)** Normal liver of control group. **(B)** CCl_4_ group with notable signs of portal fibrosis. The upper left inset depicts a higher magnification view. **(C)** CCl_4_+TAU and **(D)** CCl_4_+SIL, groups showing less extensive fibrotic areas. **(E)** CCl_4_+TAU+SIL group showing an obvious reduction in fibrosis (compared to C and D). Arrows point to fibrotic foci stained by trichrome staining (bluish color).

In Masson trichrome-stained preparations, liver sections of control group contained negligible content of collagen fibers (Fig 3A). Liver tissues of rats treated with CCl_4_ showed considerable fibrosis, characterized by expansion of collagenous tissue in portal tract (Fig 3B). By contrast, post-treatment with TAU or SIL after CCl_4_ resulted in a lower degree of fibrotic appearance (Fig 3C and 3D). The administration of TAU together with SIL resulted in greater decrease in collagen deposition than TAU or SIL-alone (Fig 3E).

### Ultrastructural pathology

**Fig 4:**
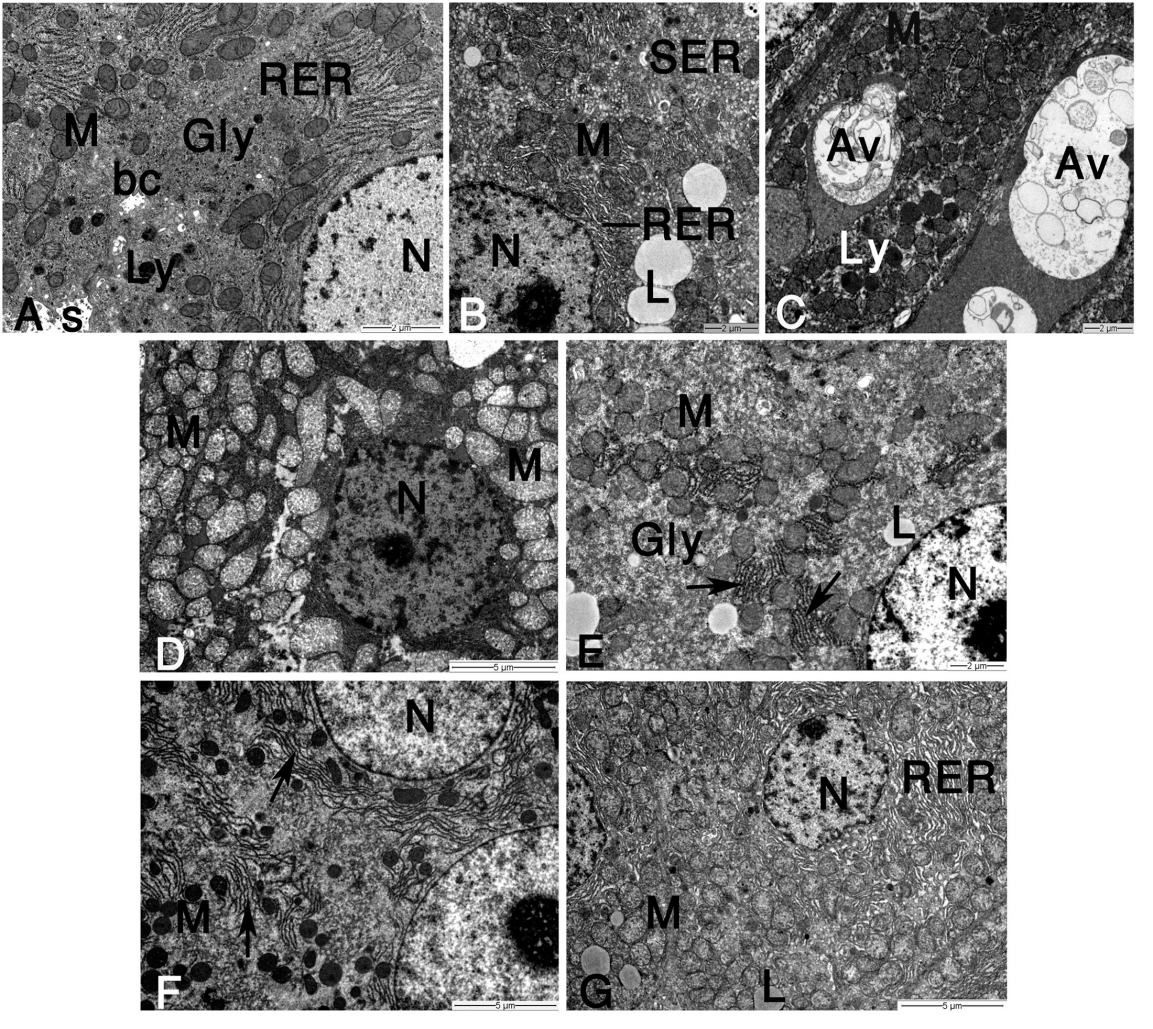
Electron micrographs of rat hepatocytes. **(A)** Control hepatocytes depicting normal architecture. Note part of nucleus (N), rough endoplasmic reticulum (RER), mitochondria (M), primary lysosomes (Ly), glycogen granules (Gly), bile canaliculus (bc), and blood sinusoid (s). **(B-D)** CCl_4_ group showing severe hepatocyte damage. Observe nucleus (N) with increased heterochromatin patches, disruption of RER membranes, proliferated smooth endoplasmic reticulum (SER), mitochondria (M) with broken cristae, large lipid droplets (L), and glycogen loss (in B). Severe reduction of RER, large autophagic vesicles and lysosomes are seen (in C). Nucleus (N) with irregular nuclear envelope and dense clumped chromatin, scanty RER membranes, and progressive mitochondrial (M) swelling are also discernible (in D). **(E)** CCl_4_+TAU group demonstrating RER fragments (arrows), mitochondria (M) with obvious cristolysis, normal glycogen rosettes (Gly), nucleus (N), and few lipid droplets (L). **(F)** CCl_4_+SIL group showing nucleus with normal chromatin content, an increase in RER cisternae (arrows) compared to CCl_4_ group, and electron-dense mitochondria (M). **(G)** CCl_4_+TAU+SIL group illustrating numerous RER profiles, normal-looking mitochondria (M), nucleus (N), and small lipid droplets (L).

Hepatocytes of control rats showed normal euchromatic nucleus, large stacks of rough endoplasmic reticulum (RER), abundant mitochondria with intact cristae, few lysosomal bodies and uniformly distributed glycogen granules in the cytoplasm (Fig 4A). Ultrastructural observation of hepatocytes in CCl_4_ group revealed extensive cellular damage. We observed a large number of fat droplets, glycogen loss, fractionation and dilatation of RER cisternal elements and abundant smooth endoplasmic reticulum (SER) arranged in large masses of closely packed vesicles (Fig 4B–4D). As shown in Fig 4C, numerous autophagosome-like vesicles and lysosomes were found within the cytoplasm of affected parenchymal cells. In most cases, mitochondria were swollen and appeared with distorted cristae and limiting membranes (Fig 4D). The nuclei were pyknotic or displayed extensive chromatin condensation, and the nuclear membrane was seen irregular, dilated or corrugated. Post-treatment of rats with TAU partially reduced the damage in hepatocytes. Some lytic areas, fat droplets, reductions in RER and mitochondrial damage were still present (Fig 4E). Also, few SER vesicles were interspersed among glycogen granules throughout the cytoplasm. In the SIL post-treated group, abnormal chromatin distribution was not revealed in the liver cells. The cytoplasm had numerous electron dense mitochondria and glycogen granules, and the integrity of the RER cisternae were preserved (Fig 4F). Hepatocytes of rats post-treated with TAU+SIL showed a normal aspect. Mitochondrial configurations were often numerous, and RER elements were dispersed, slightly reduced in number and often dilated (Fig 4G). In addition to this, most cells contained few/scattered fat droplets.

## Discussion

This study assessed the antioxidant and hepatoprotective potentials of TAU and SIL in CCl_4_-mediated hepatic oxidative injury in rats. CCl_4_ is capable of inducing hepatic injury by generating the highly reactive CCl_3_^•^ and CCl_3_OO^•^ radicals as a result of its biotransformation through the cytochrome P-450 system [5]. Both of these activated radicals bind covalently to macromolecules and initiate a chain of events leading to peroxidative degradation within membrane phospholipids and accumulation of lipid-derived oxidation products (TBARS) that cause liver damage and failure of the antioxidant defense mechanisms [7]. In the present work, the development of ultrastructural alterations in the form of disruption of RER cisternae, mitochondrial swelling and lysosomal perturbations may reflect the significantly increased hepatic TBARS levels of rats in CCl_4_ group. Post-treatment of both TAU+SIL showed stronger effects in reversing these cellular abnormalities compared to single treatments with TAU or SIL. This could be attributed to better suppression rate of TBARS and peroxides formation induced by CCl_4_. The antioxidant components system in mammalian cells have been shown to scavenge or inhibit free-radical oxygen molecules, thus protecting against CCl_4_ induced hepatopathy [9,39]. Normally, the antioxidant enzymes with GSH work in concert to detoxify superoxide anions and H_2_O_2_ in cells. SOD is a metalloenzyme which catalyzes the dismutation of superoxide anions into H_2_O_2_ and O_2_ [40]. GPx catalyzes the GSH-dependent reduction of H_2_O_2_ and hydroperoxides to non-toxic products [41]. GST is a phase II detoxification enzyme that catalyzes the conjugation of GSH with a variety of xenobiotics via cysteine thiol [42]. The non-enzymatic radical scavenger GSH is critical determinant of tissue susceptibility to oxidative damage; it a critical target for toxic oxygen and electrophilic metabolites [43]. During the enzymatic reaction catalyzed by GPx, GSH is oxidized to form GSSG which is then reduced to GSH by NADPH-dependent GR. Reduced levels of GSH play a key role in the initiation of liver necrosis [44]. In this study, intracellular GSH and antioxidants enzymes were severely depleted after CCl_4_ administration, which could lead to accumulation of ROS and oxidative hepatocellular injury [45]. TAU and SIL, separately or in combination after CCl_4_ greatly retrieved antioxidant enzymes activities towards normal control range, but SIL-alone and TAU+SIL treatments were only able to upregulate the activity of GST, as well as GSH content compared to toxin control. This increase in GSH content may be either due to *de novo* GSH synthesis, or GSH regeneration.

Oxidative stress related to CCl_4_ intoxication acts as a stimulus for fibrogenesis in experimental animals and humans [46,47]. In this study, collagen deposition in CCl_4_ group is evident from the increased level of hydroxyproline, an abundant amino acid present in collagen and also by the Masson's trichrome special staining. This was concomitant with the increased expression of TNF-α, TGF-β1, IL-6, leptin and resistin. These pro-inflammatory cytokines are largely involved in the activation of portal fibroblasts, particularly hepatic stellate cells (HSCs) which have been identified as a major collagen-producing cells in the injured liver, playing a role in the production of fibrous tissue and extracellular matrix components [48,49]. Herein, we have shown that the administration of TAU, SIL or TAU+SIL produced a significant decrease in fibrogenic markers such as TGF-β1, IL-6 and leptin while TNF-α and resistin decreased more markedly after TAU and TAU+SIL treatments. Although both TAU- and SIL-alone restored liver hydroxyproline, TAU+SIL combination was significantly more effective in reducing hydroxyproline levels. In parallel, the histopathological severity of collagen accumulation was clearly reduced in the liver with TAU+SIL combination therapy. This trend is similar to recent finding of Cao et al. [50]. Both TAU and SIL could inhibit the nuclear factor NF-_K_B which is a key regulator of inflammatory and immune reactions; they are also able to retard proliferation of HSCs [15,51,52]. Further, the present study showed that the level of circulating adiponectin in CCl_4_ model group was lower than normal, which was in agreement with the previous work of Li et al. [53]. Recent reports indicate that adiponectin plasma levels are downregulated in various liver pathological processes, including steatosis, inflammation, and fibrosis [54,55]. Again, post-treatment with TAU+SIL (but not monotherapy) reversed the decrease of adiponectin level induced by CCl_4_, probably contributing to effective reduction of hepatic inflammation and fibrogenesis by these bioactive antioxidants. To our knowledge, this is the first report of differences in adipokines production (i.e., leptin and adiponectin) associated with TAU or SIL in CCl_4_ induced subacute hepatotoxicity.

The release of TNF-α from activated Kupffer cells up-regulates iNOS and stimulates production of reactive nitrogen species (RNS) such as NO [8]. NO is known to react with superoxide anion, forming highly aggressive peroxynitrite radical, which can cause cytotoxicity and DNA damage through LPO [56]. Furthermore, iNOS may be critical in the progression of liver injury and hepatic fibrosis [57,58]. Our data demonstrated that there was an elevation in serum NO levels in CCl_4_-treated rats and that only the combined treatment of TAU+SIL was capable of attenuated serum NO levels. This may be related to the reduction of iNOS levels in hepatic tissue.

Many researchers have used AST, ALT, ALP and bilirubin as useful hallmarks of CCl_4_ hepatotoxicity [59]. Elevated levels of serum ALT and AST poisoning are indicative of cellular leakage and loss of functional integrity of membrane hepatocytes [60]. However, ALT enzyme is more specific to assess liver injury as AST is also increased following myocardial infraction and muscle injury [61]. In addition, elevated serum levels of ALP and bilirubin give a clue of hepatobiliary injury, especially interruption of the flow of bile from the liver, i.e. cholestasis [4,62]. In this study, CCl_4_ treatment caused significant elevation of serum ALT, AST, ALP and bilirubin, indicating an injury of liver cells. Conversely, TAU or SIL or both of them inhibited or modulated these serum-stimulated events and this can be further corroborated with our histopathologic analysis of liver tissue. Another marker of liver disorder is GGT, which is an enzyme catalyzing the first step in GSH degradation [63]. Increases in serum GGT as observed in CCl_4_ group can lead to an overproduction of free radicals [64]. Our data suggested that the combined treatment of TAU+SIL seemed to afford significantly more pronounced reduction of serum GGT and thus could be more beneficial than monotherapy in combating toxic radicals.

In our study, we observed an obvious increase in the levels of FFA after CCl_4_ injection in rats, which is in accordance the results reported by Ramasamy et al. [65]. The elevated levels in FFA may be in part due to oxidative lipid breakdown, which can increase the synthesis of other major lipids and activate NADPH or NADH dependent microsomal peroxidation [66]. Remarkably, the combination of TAU+SIL resulted in normalization of the FFA levels which may lead to decreased synthesis of TG, TC and extent of LPO. SIL by itself obviously exceeded TAU in lowering TG, TC and VLDL-C. SIL stimulates fatty acid β-oxidation and in turn may reduce TG biosynthesis in the liver [67]. The hypolipidemic effects of TAU are also reported in earlier studies [68]. The cholesterol lowering action of TAU was attributed to increasing the conversion of cholesterol to bile acid as a result of stimulation of 7-α hydroxylase, the rate limiting enzyme of hepatic cholesterol degradation and its conjugation to bile acids lately [69]. Moreover, it is likely possible that TAU enhanced LDL receptor binding in the liver [70], and as a result LDL-C was decreased.

In summary, the current data showed that TAU and SIL have protective effects against CCl_4_ toxicity induced liver damage, mainly via inhibition of LPO and NO production. In addition, the combination of TAU with SIL showed the best ROS/RNS-scavenging ability compared to single-drug therapy suggesting an efficient effect against inflammation and liver fibrosis. Further animal studies are required to investigate the potential of antioxidants combination (e.g., TAU+SIL) as an adjunct to liver active drugs or steroids and to elucidate their preferably pharmacological properties in liver disorders.

## Abbreviations

ALP: alkaline phosphatase
ALT: alanine aminotransferase
AST: aspartate aminotransferase
CCl_4_: carbon tetrachloride
FFA: free fatty acids
GGT: γ-glutamyltransferase
GPx: glutathione peroxidase
GR: glutathione reductase
GSH: reduced glutathione
GST: glutathione-S-transferase
HDL-C: high density lipoprotein-cholesterol
IL-6: interleukin-6
LDL-C: low density lipoprotein-cholesterol
LPO: lipid peroxidation
MDA: malondialdehyde
NO: nitric oxide
NOS: nitric oxide synthase
RER: rough endoplasmic reticulum
RNS: reactive nitrogen species
ROS: reactive oxygen species
SER: smooth endoplasmic reticulum
SIL: silymarin
SOD: superoxide dismutase
TAU: taurine
TBARS: thiobarbituric acid reactive substances
TC: total cholesterol
TG: triglycerides
TGF-β1: transforming growth factor-beta1
TNF-α: tumor necrosis factor-alpha
VLDL-C: very low density lipoprotein-cholesterol
